# mHealth and Health Information Technology Tools for Diverse Patients with Diabetes

**DOI:** 10.1155/2017/1704917

**Published:** 2017-02-23

**Authors:** Courtney R. Lyles, Neda Ratanawongsa, Shari D. Bolen, Lipika Samal

**Affiliations:** ^1^University of California, San Francisco, CA, USA; ^2^Case Western Reserve University, Cleveland, OH, USA; ^3^Harvard Medical School, Boston, MA, USA

Mobile health (mHealth) and health information technology (HIT) tools to enhance diabetes health and healthcare management have proliferated rapidly, including websites, mobile phone applications, texting or interactive voice response phone calls, remote monitoring devices/sensors, and personal health records (PHRs) linked to electronic health records [1, 2]. Many studies and systematic reviews have demonstrated that the additional communication and support provided by such technologies can improve outcomes like patient confidence, self-management, quality of life, and even health outcomes like glycemic control [3–10].

However, emerging evidence reveals a digital divide in health technology use, with lower use of widely disseminated technologies among racial/ethnic minority groups or those who have limited health literacy [11–14]. Although overall ownership and use of devices are increasing among racial/ethnic minorities, lower income individuals, and other subgroups [15, 16], there remain access, skills, and interest barriers that influence this overall digital divide [17–19]. Furthermore, research in mHealth or HIT has not often directly engaged diverse end users, as evidenced by few published studies which report that the usability of diabetes technologies among participants represents the spectrum of technological proficiency or income and educational attainment [20].

This special issue therefore provides crucial evidence about the design, testing, and implementation of mHealth or health information technology platforms for diverse patients with diabetes. The included studies cover a range of relevant research on these topics.

Two studies describe HIT-facilitated interventions to enhance diabetes self-management support by engaging both patients and their families. L. S. Mayberry et al. describe an approach to user-centered design and iterative usability testing with low-income patients to develop an mHealth intervention to promote family engagement in self-management support. Although nontechnology facilitators may be needed to engage social support networks in diabetes self-care for this population, their findings suggest preliminary feasibility for low-income patients to engage in this text messaging self-management support. Meanwhile, A. H. Lansing et al. describe the feasibility of engaging rural teens with poorly controlled type 1 diabetes and their families through an Internet-delivered intervention to improve blood glucose self-monitoring and glycemic control. Their findings prompt future research directions about sustainability and scalability, particularly to teens and families who may not be as easily incentivized in similar web-delivered self-management programs.

Two studies describe novel approaches to tailoring mHealth interventions for culture and language. V. Fontil et al. describe an approach to leveraging academic-industry partnership in developing a culturally and linguistically appropriate diabetes prevention program tailored to safety net patients with limited health literacy. While their findings are limited to a small sample drawn from an academically affiliated clinic, the approach suggests a model for engaging health technology companies in designing products that will decrease the digital divide. P. Athavale et al. describe a health coaching intervention facilitated by automated telemedicine outreach for reducing diabetes among postpartum Latina women. They address the advantages and limitations of using HIT to improve the scalability of health coaching through community organizations like local Women, Infants, and Children (WIC) Programs in an effort to reach vulnerable women at high risk of loss to follow-up.

Finally, two papers describe future directions for research in the design and implementation of online patient portals and electronic health record systems (EHRs). D. Schillinger et al. describe a research protocol to partner with computational linguistics experts to study the linguistic complexity of secure messages between diverse patients with diabetes and their healthcare teams. They propose using this novel approach to quantify and study health literacy at a population level, while also developing tools to help care teams tailor their secure message content. N. Ratanawongsa et al. describe vulnerabilities in the ways EHR electronic prescribing has affected diabetes care for diverse patients and advocate for specific changes in EHR design, implementation, policy, and research.

It is well known that many existing technological interventions have not seen wide uptake among heterogeneous settings and patient populations [21] and multifaceted, real-world research strategies that can create insights for meaningful change in the near future. Overall, we believe this special issue offers innovative approaches for including diverse populations in mHealth and health technology research and inspires future informatics, implementation, and policy researchers to build on this important work. Notably, no interventions focused on using aggregated data from mobile technology or social media to design interventions. For instance, use of aggregated data on opportunities for healthy food and safe places to be active could be used to design future public health interventions to improve the built environment. Moving forward, we must continue these multiple strands of research to truly advance the field: from discovery of new technology programs that impact health behaviors, to adaptation of existing technologies for diverse user needs, to careful consideration of implementation strategies that might differentially impact patient subgroups. Findings from these studies also indicate that policy work around increasing and sustaining low-cost broadband access will also be critical to the future success of interventions to improve care and reduce disparities using HIT.

*Courtney R. Lyles* *Neda Ratanawongsa* *Shari D. Bolen* *Lipika Samal*
